# “Living High-Training Low” improved weight loss and glucagon-like peptide-1 level in a 4-week weight loss program in adolescents with obesity: A pilot study

**DOI:** 10.1097/MD.0000000000009943

**Published:** 2018-02-23

**Authors:** Qin Yang, Guoyuan Huang, Qianqian Tian, Wei Liu, Xiangdong Sun, Na Li, Shunli Sun, Tang Zhou, Nana Wu, Yuqin Wei, Peijie Chen, Ru Wang

**Affiliations:** a Key Laboratory of Exercise and Health Sciences of Ministry of Education, Shanghai University of Sport, Shanghai, China; b Pott College of Science, Engineering and Education, University of Southern Indiana, Evansville, IN.

**Keywords:** aerobic exercise, childhood obesity, glucagon-like peptide-1, hypoxia, interleukin-6

## Abstract

**Background:**

“Living High-Training Low” (LHTL) is effective for the improvement of athletic ability; however, little is known about the effect of LHTL on obese individuals. The present study determined whether LHTL would have favorable influence on body composition, rebalance the appetite hormones, and explore the underlying mechanism.

**Methods:**

Adolescents with obesity [body mass index (BMI) >30 kg/m^2^] were randomly assigned to “Living Low-Training Low” (LLTL, n = 19) group that slept in a normobaric normoxia condition and the LHTL (n = 16) group slept in a normobaric hypoxia room (14.7% PO_2_ ∼2700 m). Both groups underwent the same aerobic exercise training program. Morphological, blood lipids, and appetite hormones were measured and assessed.

**Results:**

After the intervention, the body composition improved in both groups, whereas reductions in body weight (BW), BMI, and lean body mass increased significantly in the LHTL group (all, *P* < .05). In the LLTL group, cholecystokinin (CCK) decreased remarkably (*P* < .05) and CCK changes were positively associated with changes in BW (*r* = 0.585, *P* = .011) and BMI (*r* = 0.587, *P* = .010). However, in the LHTL group, changes in plasma glucagon-like peptide-1 (GLP-1) and interleukin-6 (IL-6) levels, positively correlated with each other (*r* = 0.708, *P* = .015) but negatively with BW changes (*r* = −0.608, *P* = .027 and *r* = −0.518, *P* = .048, respectively).

**Conclusion:**

The results indicated that LHTL could induce more weight loss safely and efficiently as compared to LLTL and increase the plasma GLP-1 levels that may be mediated by IL-6 to rebalance the appetite. Thus, an efficient method to treat obesity and prevent weight regain by appetite rebalance in hypoxia condition was established.

## 1 Introduction

Preventing and treating obesity in childhood and adolescence is crucial for a healthy adulthood.^[1]^ The most cost-effective and safe methods for the prevention and treatment of obesity include a balanced diet and aerobic exercise interventions, which have been adopted widely to reduce weight by restricting the energy intake and increasing the energy consumption.^[2,3]^ However, weight regain is a substantial challenge. Although limited studies are available on weight regain and definitions are varied in these studies, a review reported that approximately only 20% of overweight individuals are successful weight losers.^[4]^ Among various reasons for weight regain, one of the important influencing factors is increased appetite.^[5–7]^

Based on our knowledge, the regulation of appetite is rather complex. Previous studies showed that long-term (leptin, insulin) and short-term [ghrelin, peptide YY (PYY), cholecystokinin (CCK), and glucagon-like peptide-1 (GLP-1)] appetite regulating the hormones is crucial for the modulation of energy balance.^[8–11]^ Leptin and insulin reduce food intake primarily by upregulating the anorexigenic (appetite-reducing) neuropeptides and downregulating the orexigenic factors in the brain.^[12,13]^ Hitherto, ghrelin is the only hormone that has been observed to be orexigenic, whereas PYY, CCK, and GLP-1 are considered as satiety regulating hormones.^[10,11]^ These hormones respond to balanced diet and exercise-induced weight loss and play vital roles in the regulation of subjective appetite after the intervention.

Although balanced diet in combination with aerobic exercise exhibit optimal weight loss effects, increased ghrelin and decreased leptin, insulin, PYY, and CCK would reduce satiety and drive the eating desire that is unfavorable in maintaining the reduced weight.^[5,14–16]^ Moreover, another proinflammatory factor, interleukin 6 (IL-6), is reported to decrease in resting level after diet and exercise-induced weight loss process as per our previous study. IL-6 is critical in promoting food intake and satiety; thus, decreased IL-6 after weight loss may also be an adverse factor for appetite rebalance.^[17,18]^

“Living High-Training Low” (LHTL) mode has been widely used to improve athletic performance in healthy athletes based on hypoxia exposure. However, the reduced appetite and subsequent weight loss after high-altitude sojourns lead to the speculation that exposure to hypoxia may be a viable weight-reduction strategy.^[19]^ Nevertheless, only a few studies reported the effect of long-term intermittent hypoxia (IH) exposure on the increased appetite during diet and exercise-induced weight loss in individuals with obesity. Our team has designed a new randomized controlled trial to assess the effectiveness of a 4-week IH exposure plus conventional exercise training and diet intervention for inducing short- and long-term weight loss in adolescents with obesity.^[20]^ Recently, a review summarized that ghrelin, leptin, and GLP-1 seemed to be involved in long-term hypoxia exposure-induced changes in orexigenic and anorexigenic hormones.^[21]^ In addition, hypoxia would regulate IL-6 expression directly or indirectly in obesity,^[22]^ and the increased IL-6 has also been determined to reduce the food intake.^[17]^ Thus, these hormones may participate in hypoxia-mediated appetite changes. Therefore, we hypothesize that weight loss is evident, whereas satiety hormones, as well as IL-6, would be increased after LHTL intervention than “Living Low-Training Low” (LLTL). Furthermore, we compared the changes in body composition, blood lipids, and appetite hormones at the baseline level and postintervention between subjects who slept in normoxia and hypoxia condition to explore the mechanism underlying LHTL on weight loss and appetite regulation.

## 2 Methods

### 2.1 Study design

**Figure 1:**
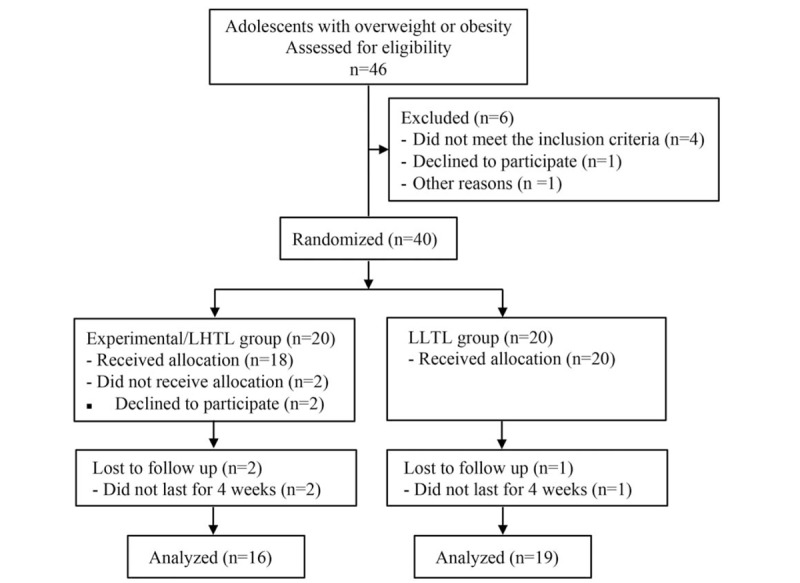
Schematic of the study design. LHTL = “Living High-Training Low” group, LLTL = “Living Low-Training Low” group.

The study, comprising of 35 adolescents, was a randomized, assessment-blinded, controlled clinical trial spanning over 4 weeks. A public health nurse assessed the Tanner stage of each subject using the Tanner grading system.^[23,24]^ A criterion of body mass index (BMI)-matched age of male and female children with obesity was fulfilled for inclusion in the study.^[25]^ The trial was registered with the Chinese Clinical Trial Registry (ChiCTR-TRC-14004106). This trial adhered to the tenets of the Declaration of Helsinki. The protocol and informed consent were approved by the institutional review board of Shanghai University of Sport, Shanghai, China. Written informed consents were obtained from all participants and their parents. Because the subjects were obese children and adolescents and need to sleep in a normobaric normoxia condition, we first talked to subjects and their parents about the principles of hypoxia intervention, the detailed process of sleeping in normobaric normoxia condition, the potential risks and the possible physical effects so that they fully understand the experimental process and decide whether to participate or not. Then, subjects who volunteered to participate in the experiment would be scheduled to sleep in normobaric normoxia condition for 1 night to decide whether discomfort problems occurred and whether to continue to participate in the experiment. All participants received identical well-defined and balanced diet program daily during the intervention. The study employed a physician to ensure the health eligibility and safety of all participants. Figure 1 provided an overview of the study protocol.

### 2.2 Eligibility criteria for participants

The eligibility criteria for participation in the study were as follows: participants should be 12- to 16-year-old with BMI values greater than the international standard definition of age- and sex-specific BMI for adolescents with obesity.^[25]^ The subjects were excluded from the study if they presented concomitant renal, hepatic, cardiac disease, frequently participated in physical activities, structured exercise, nutrition intervention, weight loss programs, and/or were being treated with medications that could affect the body weight (BW) and appetite within 6 months before the initial screening.

### 2.3 Recruitment of participants

The study participants were children who registered for the summer weight loss camp at Shanghai University of Sport, Shanghai, China, where they were provided systematic medical, psychological, and nutritional assistance. The recruitment period was May 1 to August 1, 2014. Initially, we randomized 46 subjects and a cohort of 40 qualified for the study (18 girls) [mean ± standard deviation (SD); age = 14.1 ± 1.5 years]. Then, they were randomly assigned to LLTL (control) and LHTL groups. Two adolescents in the LHTL group dropped out of the study for personal reasons. Two adolescents in the LHTL group and 1 in the LLTL group did not complete the 4-week intervention period for collection of anthropometry data and blood indicators; therefore, 16 children in the LHTL group (8 girls, mean ± SD; age = 14.3 ± 1.4 years; BMI = 32.9 ± 3.5 kg/m^2^) and 19 in the LLTL group (8 girls, mean ± SD; age = 13.9 ± 0.9 years; BMI = 31.5 ± 3.4 kg/m^2^) completed the intervention. The sample size was estimated using the G∗Power software 3.1.9.2 (Universität Düsseldorf)^[26]^ considering a statistical power (1 – β) of 0.8, effect size of 0.3, and *P* *=* .05. The minimal sample size required for each study group was 8.

### 2.4 Randomization

The randomization was performed using the individual camping numbers. All participates eligible according to the inclusion criteria were allocated in either the LLTL (control) or the LHTL (experimental) groups by the EXCEL-based random number table. The subjects’ camping number was blindly entered into the EXCEL random number table and termed as “LLTL” and “LHTL.” Twenty numbers were allocated to each group and generated LLTL and LHTL groups according to the allocation of the camping number.

### 2.5 Intervention

#### 2.5.1 Exercise training

Both LLTL and LHTL groups applied the same aerobic exercise program according to our previous methods.^[18]^ All participants underwent an intense exercise program (6 days/week, 2 times daily, 2 h/session), which consisted of swimming [intensity: 6 Metabolic Equivalent of Task (MET)], aerobic exercise (intensity: 7.5 MET), and basketball (intensity: 6 MET). The MET values were established using pulmonary function equipment according to the manufacturer's instructions (Cosmed, K4b2, Roma, Italy). The oxygen consumption (VO_2_) for all activities was measured using the Cosmed K4b2 portable metabolic system. The exhaled respiratory gases were collected on a breath-by-breath basis during a submaximal treadmill (H/P/Cosmos Pulsar 4.0, Nussdorf-Traunstein, Germany) test. The participants began the exercise at a speed of 2 km/h, increased by 1 km/h every 2.5 minutes until 8 km/h was reached, without slope gradient in the treadmill. Eighty percent of the maximum heart rate (HR_max_, [220-age]) was set as a standard of exercise termination, during which VO_2_ and HR data were collected. Trained research assistants recorded the heart rate and power output data at the end of each stage. Based on these values, individual linear regression equations were developed for predicting the VO_2_.^[27]^ Oxygen uptake values (Ml · kg^−1^ · min^−1^) were converted to units of energy expenditure (MET) by dividing by 3.5 (1 MET is defined as the resting metabolic rate, which consumes 3.5 mL O_2_/min). The heart rate was measured by fingertip pulse oximeter every 15 to 30 minutes during the exercise for each individual to ensure the exercise intensity within the range of target heart rate [target heart range = resting heart rate + (220 − resting heart rate) × (20%–40%)].

#### 2.5.2 Hypoxic exposure

Subjects in the LHTL group slept in a normobaric hypoxic environmental chamber every night during the intervention. A large hypoxic training system (TOSMA, International Hypoxia-Trainings Center, Berlin, Germany) in the Hypoxia Test Laboratory of Shanghai Oriental Oasis Training Base was used to simulate the hypoxic environment (14.7% O_2_; ∼2700 m) that can control or minimize the influence of other confounding factors, such as temperature, humidity, and physical activity levels, presented in real altitude situation. After 24 hours hypoxia acclimation, the participants were arranged to sleep in the chamber for 10 hours every night (from 21:00–7:00 am on the following day), 7 days/week, for 4 weeks.

### 2.6 Measurement

All measurements were performed 2 days before and after the intervention. The anthropometry and body composition included BW, BMI, lean body mass (LBM), total fat mass (TFM), and body fat percentage (BFP). The blood indicators included leptin, insulin, ghrelin, PYY, CCK, GLP-1, and circulating levels of IL-6 in 12-hour fasting status. All assessments were performed at the Laboratory of Exercise Physiology at the Shanghai University of Sport.

#### 2.6.1 Anthropometry and body composition

BW and height were measured using a digital scale (TANITA, Tokyo, Japan). The body composition and fat distribution were measured using dual energy x-ray absorptiometry (DEXA) (GE Lunar Prodigy, Fairfield, CT). The ENCORE software (version 10.50. 086) was used to analyze LBM, TFM, and BFP.

#### 2.6.2 Blood analyses

Fasting blood samples were obtained at baseline and postintervention. Following manufacturers’ instructions, we evaluated the blood biochemistry including fasting blood glucose (FBG), plasma triglycerides (TG), total cholesterol (TC), and high-/low-density lipoprotein (HDL or LDL) by enzyme-linked immunosorbent assay (ELISA) (ELISA kits purchased from HOMA Biological Engineering Co., Ltd, Beijing, China). Absorbance was measured at 450 nm using a microplate reader (Bio-Rad 550, Hercules, CA). PYY, CCK (R&D systems, MN), leptin, insulin, ghrelin, GLP-1, and IL-6 were measured by the suspension array system (Bio-Plex 200 Laboratories Inc, CA).

### 2.7 Statistical analysis

Statistical analyses were performed using statistical package SPSS (version 19.0, Armonk, NY). Data normality of all variables was confirmed by Kolmogorov–Smirnov test, and the results were expressed as mean ± SD. Independent *t* tests and chi-square analyses were conducted to compare the continuous variables and qualitative data at baseline, respectively. One-way analysis of variance with repeated measures and a Tukey post hoc test was applied to see if there was significant difference for all biomarkers associated with anthropometry, body composition, and blood lipid indicators between the baseline and post-intervention. Pearson's correlation analyzed the relationship between the changes in body composition and appetite hormones following the intervention. Significance was set at a 2-tailed *P* value <.05.

## 3 Results

### 3.1 Basic characteristics of the 2 groups

Thirty-five subjects (19 in the LLTL and 16 in the LHTL group) were included in the final analysis. Table 1 displayed the baseline characteristics of subjects before the intervention. No significant differences were observed between the 2 groups in terms of BW, BMI, LBM, TFM, BFP, blood glucose homeostasis [FBG, insulin, homeostasis model assessment of insulin resistance (HOMA2-IR)], and blood lipids (TG, TC, HDL/LDL). However, a higher fasting plasma insulin level was found in the LHTL group as compared to that of the LLTL group (*P* < .05). These phenotypes reflected a differential ability of pancreatic beta cells in response to stimuli among the individuals. Moreover, FBG and HOMA2-IR did not show any differences between the 2 groups. Therefore, it was speculated that no differences occurred in IR or glucose intolerance between the 2 groups.

**Table 1:**
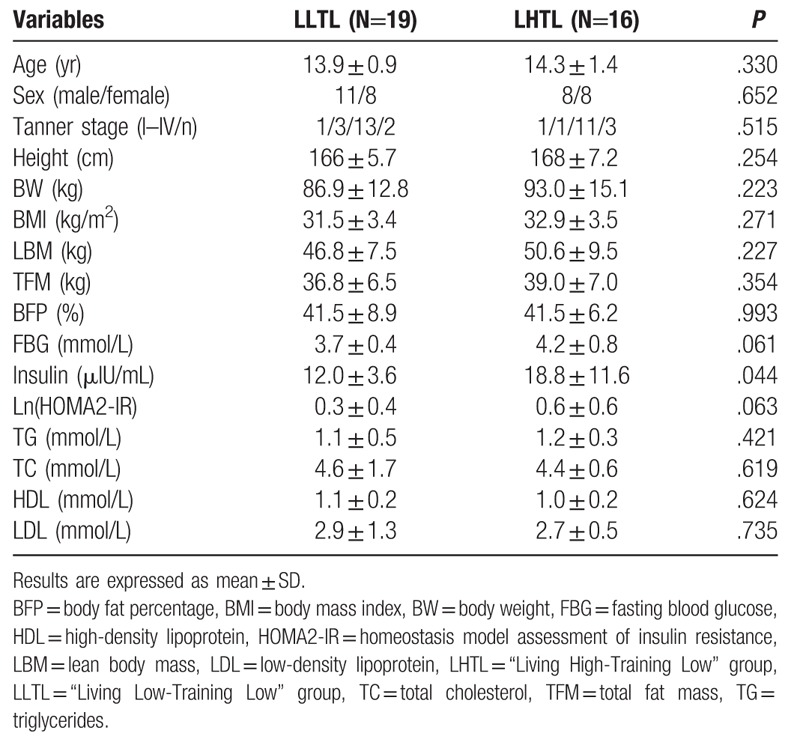
Baseline characteristics of the subjects.

### 3.2 Changes in body weight, body composition, and blood indicators

**Figure 2:**
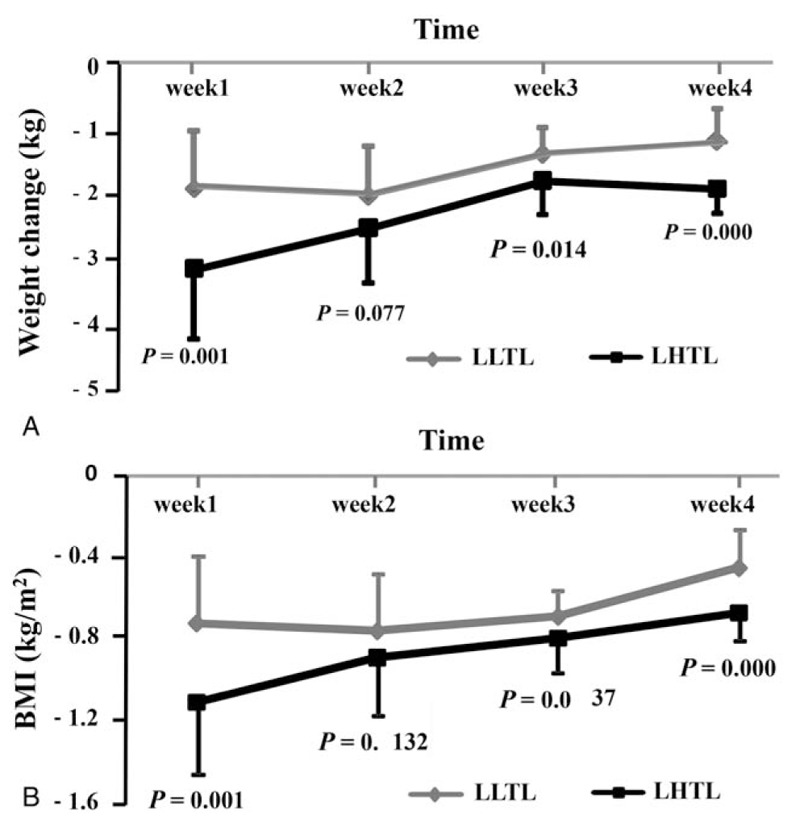
Weekly changes in the weight and body mass index (BMI) between the 2 groups. Error bar indicates mean ± SD. Changes in body weight (BW) (A) and BMI (B) during the 4-week intervention in LLTL and LHTL groups. LHTL = “Living High-Training Low” group, LLTL = “Living Low-Training Low” group. *P* value represents the comparison of variables in each week between LLTL and LHTL groups during the intervention period.

We first analyzed the anthropometry and body composition indicators. As shown in Figure 2, BW and BMI showed maximum and minimum reductions in the first and fourth weeks, respectively. Table 2 displayed that BW, BMI, TFM, and BFP decreased significantly after the 4-week intervention in each group as compared to their baseline levels (all, *P* < .01), whereas LBM in the LHTL group reduced significantly after intervention (*P* < .05). In order to avoid the deviation in the values of BW and body composition indicators, we compared the changes in these indicators between groups. Notably, the altered BW, BMI, and LBM in the LHTL group displayed a more significant effect as compared to the LLTL group (*P* < .05, *P* < 0.05, and *P* < .05).

**Table 2:**
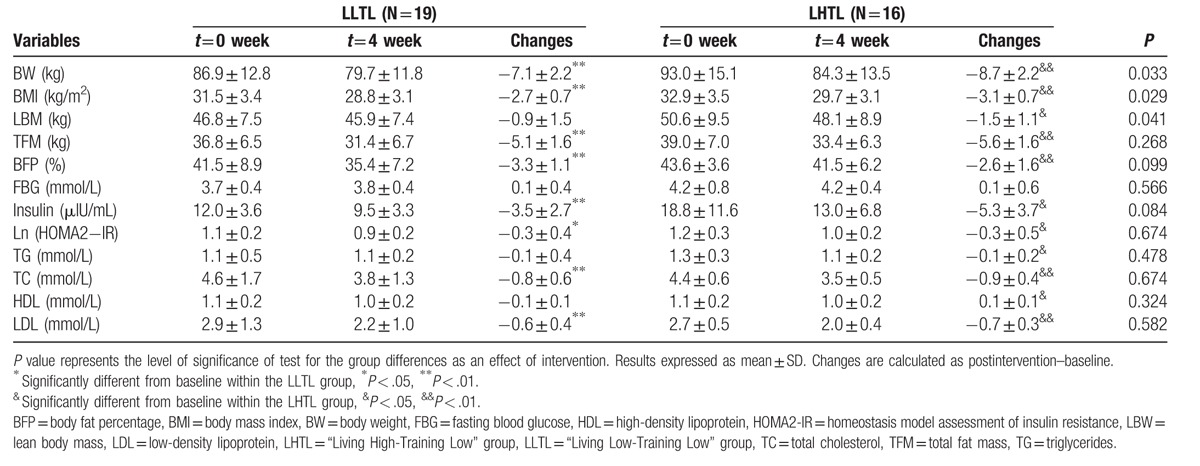
Changes in morphology, blood glucose, and lipid indicators.

With respect to blood indicators, FBG did not exhibit significant changes after intervention in each group. However, the plasma insulin levels and HOMA2-IR decreased significantly after intervention in the LLTL (*P* < .01 and *P* < .05) and LHTL groups (*P* < .05 and *P* < .05); the data may indicate an improved insulin resistance and glucose uptake function. TC and LDL decreased significantly in each group as compared to their baseline levels (*P* < .01 and *P* < .01). In addition, a significant decrease in TG (*P* < .05) and increase in HDL (*P* < .05) was found in the LHTL group. However, the changes in the blood lipid indicators did not vary significantly between the 2 groups after intervention.

### 3.3 Changes in appetite hormones

#### 3.3.1 Long-term appetite hormones

**Figure 3:**
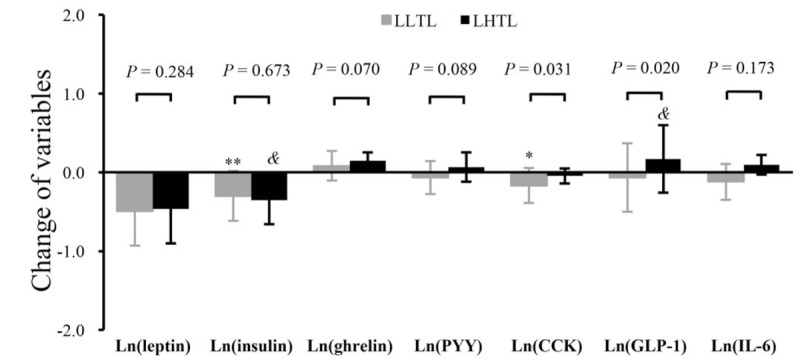
Change in appetite hormones before and after intervention between the 2 groups. Results are expressed as mean [Ln(post-intervention)-Ln(baseline)] ± SD [Ln(post-intervention)-Ln(baseline)]. LHTL = “Living High-Training Low” group, LLTL = “Living Low-Training Low” group. ^∗^Significantly different from baseline within the LLTL group, ∗*P* < .05, ∗∗*P* < .01; ^&^indicates significantly different from baseline within the LHTL group, ^&^*P* < .05; *P* value represents the level of significance of the test for the group differences in the effect of the intervention.

After the intervention, the insulin levels reduced significantly in LLTL (*P* < .01) and LHTL groups (*P* < .05) (Fig. 3), whereas leptin levels did not show significant changes as compared to the baseline levels in each group. However, the changes in leptin and insulin did not differ significantly between the 2 groups.

#### 3.3.2 Short-term appetite hormones and interleukin-6

The baseline levels of GLP-1, PYY, CCK, ghrelin, and IL-6 did not show any significant difference between the 2 groups. After the intervention, a significant decrease was noted in CCK (*P* < .05) in the LLTL group and a significant increase in GLP-1 (*P* < .05) in the LHTL group (Fig. 3). Moreover, the changes in CCK (*P* < .05) and GLP-1 (*P* < .05) were found to significantly different between the 2 groups. None of the other appetite hormones showed significant changes after intervention as compared to preintervention in each group or presented significant differences between the LLTL and LHTL groups.

### 3.4 Correlation between changes in appetite hormones and body weight, body composition, and blood lipids

**Figure 4:**
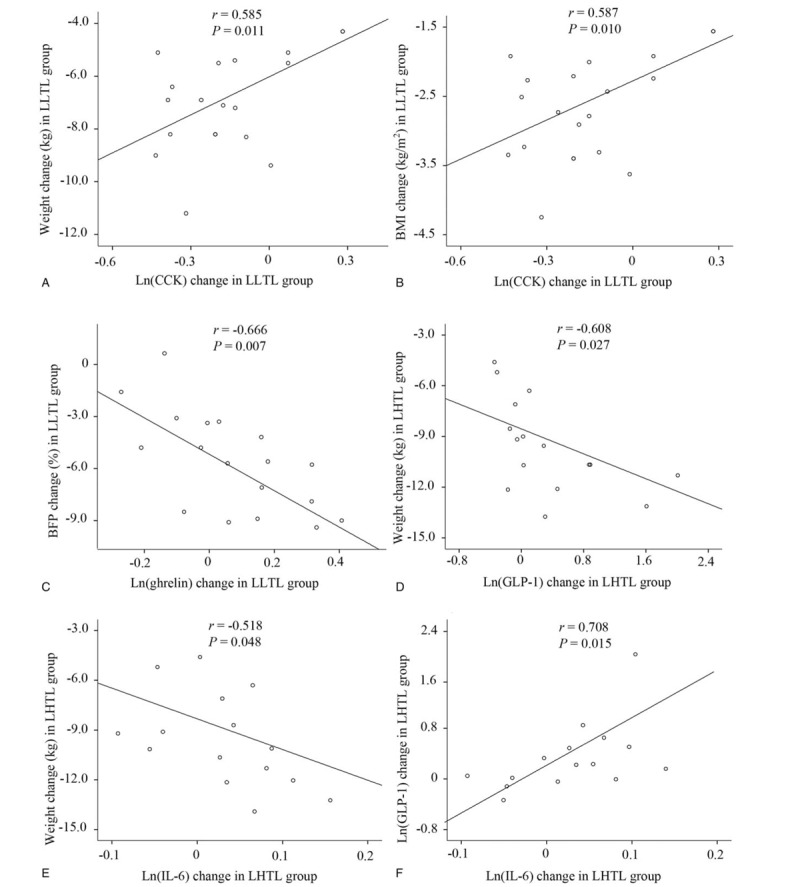
Correlation between appetite-related hormones and weight change, body mass index (BMI), and body fat percentage (BFP) in the 2 groups. Pearson's correlation analysis showed that in the LLTL group, Ln(CCK) changes were positively correlated with weight change (A) and BMI change (B), respectively, and Ln(ghrelin) change was negatively correlated with BFP change (C). In contrast, in the LHTL group, Ln(CCK) change was positively correlated with BMI change (D), whereas Ln(IL-6) change was negatively correlated with weight change (E) and positively correlated with Ln(GLP-1) change (F). CCK = cholecystokinin, GLP-1 = glucagon-like peptide-1, IL-6 = interleukin-6, LHTL = “Living High-Training Low”, LLTL = “Living Low-Training Low” group.

Pearson's correlation analysis revealed that changes in the levels of leptin and insulin did not correlate significantly with changes in the above morphological and glucolipid variables in both LLTL and LHTL groups after intervention. As shown in Figure 4A–C, altered CCK in the LLTL group was positively associated with weight change (*r* = 0.585, *P* = .011) and BMI change (*r* = 0.587, *P* = .010), whereas altered level of ghrelin was negatively correlated with BFP (*r* = −0.666, *P* = .007). In addition, CCK reduction was positively correlated with reduced TG (*r* = 0.634, *P* = .005), TC (r = 0.613, *P* = .0057), HDL (*r* = 0.569, *P* = .014), and LDL (*r* = 0.499, *P* = .035), respectively (data not shown). Conversely, the weight change in the LHTL group was negatively correlated with GLP-1 (*r* = −0.608, *P* = .027) and IL-6 (*r* = −0.518, *P* = .048) (Fig. 4D–E). Moreover, a positive association was observed in the changes between GLP-1 and IL-6 in the LHTL group (Fig. 4F, *r* = 0.708, *P* = .015).

## 4 Discussion

In the present study, we found that LHTL intervention reduced more BW improved blood chemistry and glucolipid after intervention in both groups. In the LHTL group, GLP-1 levels rose greatly after intervention, and weight change was closely related to the changes in GLP-1 and IL-6, indicating an upward trend of satiety hormones. Moreover, changes in GLP-1 and IL-6 levels were positively correlated, implying an IL-6/GLP-1 synergistic effect in the LHTL group. This finding allows us to first propose that LHTL might induce the upregulation of GLP-1 that may be mediated by IL-6. Consequently, changes in both satiety factors after intervention could be beneficial for the appetite regulation during long-term weight management in adolescents with obesity. Thus, our results proposed a method that may be effective and practical for weight loss practice through exercise in normal conditions and sleeping in hypoxic conditions.

Interventions with hypoxic exposure are effective in reducing the weight. Presently, most studies have focused on the effect of short-term passive IH exposure or active hypoxic training in healthy subjects. However, we recruit adolescents with obesity as the subjects and use an intervention that distinguish the hypoxia intervention from exercise training process, which simultaneously avoids the load of exercise and hypoxic effects on individuals with obesity. Furthermore, the intervention would coordinate the body's response to hypoxia and exercise in an effective and operable manner. The current study demonstrated that LHTL for 4 weeks might produce favorable cumulative benefits on both weight loss, lipids metabolism, and appetite hormones. These results provided convincing evidence that LHTL intervention exerted favorable benefits on individuals with obesity than LLTL.

Among the beneficial effects of hypoxia on the body, the changes in appetite hormones during hypoxic exposure are varied. Some studies revealed that changes in ghrelin and CCK were supposedly responsible for anorexia at high altitude,^[28,29]^ whereas others were identified decreased or unaffected satiety hormones during hypoxic exposure.^[30,31]^ The current results indicated a decrease in CCK levels in the LLTL but not the LHTL group, thereby indicating a reduced satiety after LLTL intervention. Based on the background that leptin and insulin are elevated in obesity and differently affected by hypoxia and aerobic exercise,^[32–34]^ we may speculate that the effect of aerobic exercise and hypoxia on leptin and insulin regulation is benign and the changes are accompanied by weight loss. Thus, leptin and insulin displayed a decreasing trend or a significant decrease in both groups, whereas significance between the groups was not found. Moreover, these changes would be favorable for improved sensitivity of leptin and insulin and energy homeostasis of whole body, as well as, individuals with obesity, although crucial long-term appetite regulators are present. The differential regulation of leptin and insulin in response to exercise and hypoxia necessitates further research to explore the mechanism underlying the combination of “aerobic exercise” or “sleeping in hypoxia” in LHTL on leptin and insulin secretion and function.

We also found an increase in GLP-1 levels in the LHTL group as compared to the LLTL group. The result leads us to explore the role of hypoxia in GLP-1 regulation. Studies on healthy subjects showed that short-term or long-term hypoxia exerted a limited influence on GLP-1 levels,^[30,35]^ whereas long-term hypoxia and inactivity caused a decrease in the levels of postprandial GLP-1.^[30]^ However, studies on the effect of long-term hypoxia exposure on GLP-1 regulation in individuals with obesity are scarce. Moreover, GLP-1 has been shown to promote satiety, suppresses energy intake, and prevent weight gain.^[36,37]^ Individuals with obesity showed up to 20% impaired GLP-1 response as compared to normal weight individuals.^[38]^ Consistently, we found a significant decrease in the BW, which positively correlated with an evident increase in GLP-1 levels after LHTL intervention. Thus, the increased GLP-1 levels may indicate a beneficial trend for controlling the BW. However, the specific mechanism underlying hypoxia-mediated regulation of GLP-1 levels warrants further studies.

Notably, the inflammatory factor, IL-6, showed an increased trend but not to a significant level, and presented a negative correlation with weight change in subjects with LHTL intervention. This phenomenon was enhanced by exercise or other factors, and IL-6 has been reported to induce weight loss and alleviate obesity-induced fatty liver and insulin resistance.^[39,40]^ Moreover, the disruption of hypothalamic-specific IL-6 activity could block the beneficial effects of exercise on BW and/or the rebalance of food intake and insulin and leptin resistance.^[17]^ Previous studies reported that hypoxia exposure might promote the secretion of IL-6 in adipocytes, and thus, may effectuate the metabolism through several mechanisms.^[22,41]^ In this study, the 4-week LHTL intervention induced significant weight loss and a corresponding increase in IL-6 level. The negative correlation between alterations in IL-6 change and weight implies a putative rebalanced appetite in an IL-6-dependent response postintervention.

Interestingly, we found a positive relationship between changes in GLP-1 and IL-6 in the LHTL group. GLP-1 and IL-6 have been reported to interact with each other. IL-6 may reduce the inflammation and insulin resistance in a GLP-1-dependent manner,^[42,43]^ whereas GLP-1 may affect the food intake and weight change in an IL-6-dependent manner.^[44]^ Although hypoxia activates IL-6 and GLP-1 signal, how hypoxia activates the IL-6/GLP-1 signal and the effect of different oxygen concentration on IL-6/GLP-1 signal modulation is yet unknown. Herein, we may infer that hypoxia-induced significant secretion of GLP-1 may be partially mediated by IL-6, a regulator that responds directly to hypoxia through NF-κB activation.^[45]^ However, the mechanism of appetite regulation is sophisticated and might be involved in synergistic or antagonistic effects of different interventions such as exercise, diet, and hypoxia. Thus, whether the LHTL-induced weight loss and appetite re-balance would be partially mediated by the synergies of IL-6 and GLP-1 is yet to be elucidated. The mechanism underlying the changes in IL-6 and GLP-1 signal occur in central and peripheral organs after long-term intervention necessitate further investigations. Whether these changes result in different effects of hypoxia, diet, and exercise intervention on the regulation of appetite is also worth exploring further.

Obesity is a risk factor for apnea syndrome. Although the effect in adults of hypoxia intervention is fairly known, there is scarce research in literature about safety of this intermittent exposure to hypoxic conditions in children and adolescents.^[46]^ Previous reviews summarized the usefulness of moderate IH conditioning/training in sick children for treating their various forms of disease but also emphasized the adverse effects that should be noted.^[47,48]^ However, we did not find any subject with discomfort symptoms (hypoxia-induced symptoms such as pulmonary vasoconstriction, hyperventilation, cerebrovascular relaxation, sympathetic nervous activity, etc) during hypoxia intervention period. We have taken this problem into full consideration. Each participant underwent rigorous screening of cardiovascular and other non-applicable conditions before the inclusion to ensure safety, and professional sports physician also paid close attention to individuals during hypoxia intervention periods and focused on the subject's reaction to consider whether he or she was appropriate to continue the experiment. In fact, most of the lung ventilation index and basic metabolic variables were improved after both interventions, indicating their efficiency and safety in hypoxic treatment (14.7% O2; ∼2700 m) of obese adolescents for weight loss and management. Moreover, a mean change in weight of 7 kg obtained after both interventions in a month was striking but achievable for obese adolescents, which further proved the effectiveness of the 2 interventions.^[18,49]^ Notably, this study presented some limitations. First, this is a single-center study with small sample size and did not achieve double-blindness. Second, the short intervention time may also be a confounding factor for the inconspicuous change in appetite hormones. Third, evaluation of the objective appetite is scarce; the subjective appetite of a majority of the weight losers showed an increasing trend after intervention in our previous survey results. The subjects in this summer camp would return to all over the country; thus, data on weight regain and safety assessment should be collected by further follow-ups. Therefore, appropriate follow-up studies are essential for long-term weight management through the LHTL method.

## 5 Conclusion

In conclusion, our study demonstrated that LHTL might have a better effect than LLTL on managing the BW and composition in adolescents with obesity. It can induce a favorable weight loss and alter the appetite hormones for the benefit of the long-term weight management through a mechanism of upregulation of GLP-1, a satiety hormone that may be regulated by IL-6 for appetite re-balance during the long-term intervention.

## Acknowledgments

The authors thank the study participants and other at the “Shanghai Dianfeng Weight Management Limited” for providing the staff, sports venues, sports, and nutrition monitoring.
